# 2-Benzyl­oxybenzaldehyde azine

**DOI:** 10.1107/S1600536809046728

**Published:** 2009-11-11

**Authors:** Fei-Fei Cen, Chen Xu, Zhi-Qiang Wang, Lin Cheng, Yu-Qing Zhang

**Affiliations:** a Chemical Engineering and Pharmaceutics School, Henan University of Science and Technology, Luoyang 471003, People’s Republic of China; b College of Chemistry and Chemical Engineering, Luoyang Normal University, Luoyang 471022, People’s Republic of China

## Abstract

The complete mol­ecule of the title compound, C_28_H_24_N_2_O_2_, is generated by a centre of inversion (at the mid-point of the N—N bond). The substituents at the ends of the C=N bonds adopt an *E*,*E* configuration. The central –CH=N—N=CH– fragment is planar, but as a whole the mol­ecule is not: the benz­yloxy group is rotated about the O—C bond by 69.3 (2)° with respect to the plane of the benzyl­idene hydrazine unit.

## Related literature

For general background to the coordination capability and biological activity of Schiff bases, see: Amadei *et al.* (1998 ▶); Xu *et al.* (2007 ▶). For related structures, see: Glidewell *et al.* (2006 ▶); Chattopadhyay *et al.* (2008 ▶). For the synthesis, see: Fu (2007 ▶).
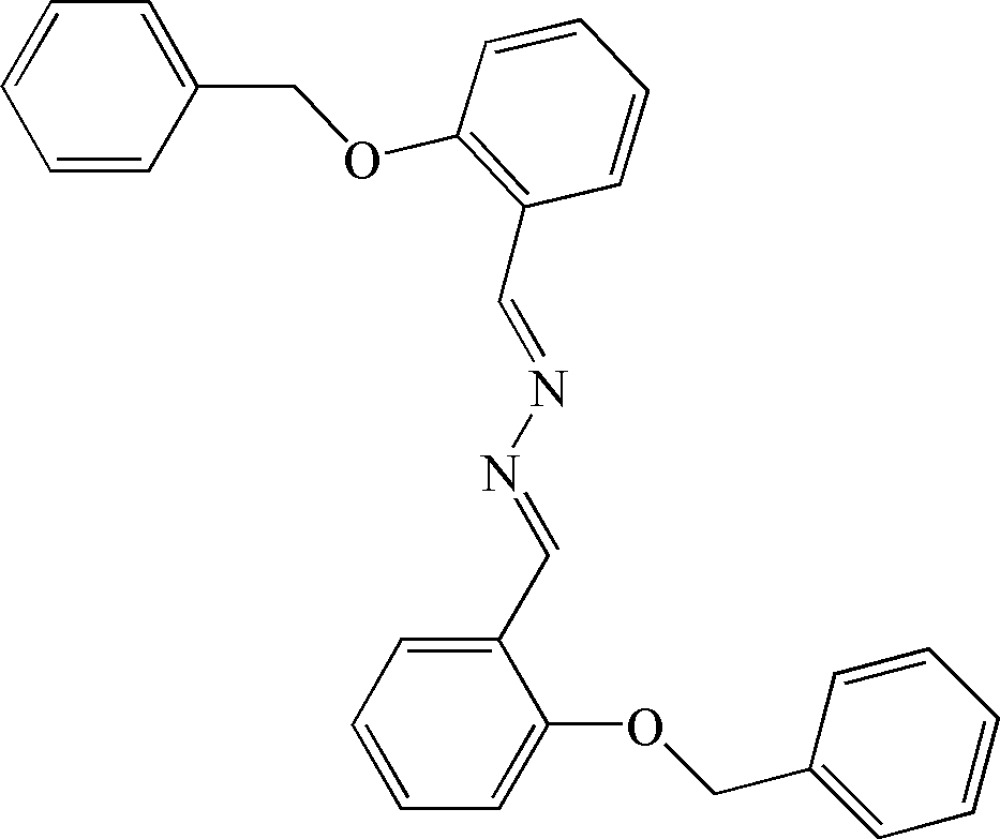


## Experimental

### 

#### Crystal data

C_28_H_24_N_2_O_2_
*M* *_r_* = 420.49
Monoclinic, 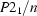

*a* = 11.222 (2) Å
*b* = 8.1157 (15) Å
*c* = 12.799 (2) Å
β = 102.297 (3)°
*V* = 1138.9 (4) Å^3^
*Z* = 2
Mo *K*α radiation
μ = 0.08 mm^−1^
*T* = 294 K
0.30 × 0.22 × 0.06 mm

#### Data collection

Bruker SMART APEX CCD area-detector diffractometer
Absorption correction: none
8428 measured reflections
2125 independent reflections
1143 reflections with *I* > 2σ(*I*)
*R*_int_ = 0.049

#### Refinement

*R*[*F* ^2^ > 2σ(*F* ^2^)] = 0.047
*wR*(*F* ^2^) = 0.119
*S* = 1.02
2125 reflections
146 parameters
H-atom parameters constrained
Δρ_max_ = 0.12 e Å^−3^
Δρ_min_ = −0.12 e Å^−3^

### 

Data collection: *SMART* (Bruker, 2004 ▶); cell refinement: *SAINT* (Bruker, 2004 ▶); data reduction: *SAINT*; program(s) used to solve structure: *SHELXS97* (Sheldrick, 2008 ▶); program(s) used to refine structure: *SHELXL97* (Sheldrick, 2008 ▶); molecular graphics: *SHELXTL* (Sheldrick, 2008 ▶); software used to prepare material for publication: *SHELXTL*.

## Supplementary Material

Crystal structure: contains datablocks global, I. DOI: 10.1107/S1600536809046728/rn2064sup1.cif

Structure factors: contains datablocks I. DOI: 10.1107/S1600536809046728/rn2064Isup2.hkl

Additional supplementary materials:  crystallographic information; 3D view; checkCIF report

## Figures and Tables

**Fig. 1.**
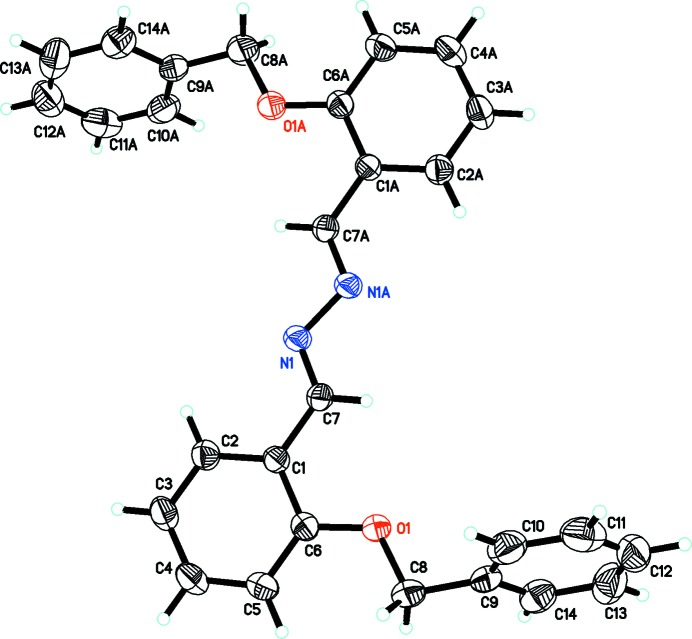
The molecular structure of the title compound with displacement ellipsoids at the 30% probability level (suffix A denotes the symmetry code: -x + 2, -y, -z).

## supplementary crystallographic information

### Comment

Schiff bases have received much attention during the past decades because of their strong coordination capability and diverse biological activities (Amadei *et al.*, 1998; Xu *et al.*, 2007). Among them, azines are obtained from the condensation of an aldehyde or ketone with hydrazine.

The title compound possesses a crystallographically imposed center of symmetry at the midpoint of the N—N bond (Fig.1). It adopts an *E*, *E* configuration, which is similar to those of the related compounds (Glidewell, *et al.*, 2007; Chattopadhyay *et al.*, 2008). The C7—N1 [1.266 (2) Å] and N1—N1A [1.414 (3) Å] distances indicate these correspond to double and single bonds, respectively. The central –CH=N—N=CH– fragment is planar, but as a whole the molecule is not planar. The benzyloxy group is rotated about the O—C bond by 69.3 (2)° with respect to the plane of the benzylidene hydrazine moiety.

### Experimental

The title compound was prepared as described in literature (Fu, 2007) and recrystallized from ethanol at room temperature to give the desired crystals suitable for single-crystal X-ray diffraction.

### Refinement

H atoms attached to C atoms of the title compound were placed in geometrically idealized positions and treated as riding with C—H distances constrained to 0.93–0.96 Å, and with *U*_iso_(H)=1.2–1.5*U*_eq_(C).

### Crystal data

**Table:**

C_28_H_24_N_2_O_2_	*F*(000) = 444
*M**_r_* = 420.49	*D*_x_ = 1.226 Mg m^−^^3^
Monoclinic, *P*2_1_/*n*	Mo *K*α radiation, λ = 0.71073 Å
*a* = 11.222 (2) Å	Cell parameters from 930 reflections
*b* = 8.1157 (15) Å	θ = 2.7–20.6°
*c* = 12.799 (2) Å	µ = 0.08 mm^−^^1^
β = 102.297 (3)°	*T* = 294 K
*V* = 1138.9 (4) Å^3^	BLOCK, yellow
*Z* = 2	0.30 × 0.22 × 0.06 mm

### Data collection

**Table:**

Bruker SMART APEX CCD area-detector diffractometer	1143 reflections with *I* > 2σ(*I*)
Radiation source: fine-focus sealed tube	*R*_int_ = 0.049
graphite	θ_max_ = 25.5°, θ_min_ = 2.7°
φ and ω scans	*h* = −13→13
8428 measured reflections	*k* = −9→9
2125 independent reflections	*l* = −15→15

### Refinement

**Table:**

Refinement on *F*^2^	Secondary atom site location: difference Fourier map
Least-squares matrix: full	Hydrogen site location: inferred from neighbouring sites
*R*[*F*^2^ > 2σ(*F*^2^)] = 0.047	H-atom parameters constrained
*wR*(*F*^2^) = 0.119	*w* = 1/[σ^2^(*F*_o_^2^) + (0.043*P*)^2^ + 0.1257*P*] where *P* = (*F*_o_^2^ + 2*F*_c_^2^)/3
*S* = 1.02	(Δ/σ)_max_ < 0.001
2125 reflections	Δρ_max_ = 0.12 e Å^−^^3^
146 parameters	Δρ_min_ = −0.12 e Å^−^^3^
0 restraints	Extinction correction: *SHELXL97* (Sheldrick, 2008), Fc^*^=kFc[1+0.001xFc^2^λ^3^/sin(2θ)]^-1/4^
Primary atom site location: structure-invariant direct methods	Extinction coefficient: 0.015 (3)

### Special details

**Table:**

Geometry. All e.s.d.'s (except the e.s.d. in the dihedral angle between two l.s. planes)are estimated using the full covariance matrix. The cell e.s.d.'s are takeninto account individually in the estimation of e.s.d.'s in distances, anglesand torsion angles; correlations between e.s.d.'s in cell parameters are onlyused when they are defined by crystal symmetry. An approximate (isotropic)treatment of cell e.s.d.'s is used for estimating e.s.d.'s involving l.s. planes.
Refinement. Refinement of *F*^2^ against ALL reflections. The weighted *R*-factor *wR* andgoodness of fit *S* are based on *F*^2^, conventional *R*-factors *R* are basedon *F*, with *F* set to zero for negative *F*^2^. The threshold expression of*F*^2^ > σ(*F*^2^) is used only for calculating *R*-factors(gt) *etc*. and isnot relevant to the choice of reflections for refinement. *R*-factors basedon *F*^2^ are statistically about twice as large as those based on *F*, and *R*-factors based on ALL data will be even larger.

### Fractional atomic coordinates and isotropic or equivalent isotropic displacement parameters (Å^2^)

**Table:**

	*x*	*y*	*z*	*U*_iso_*/*U*_eq_	
C1	0.76645 (18)	0.1813 (2)	−0.07028 (15)	0.0456 (5)	
C2	0.7692 (2)	0.2275 (3)	−0.17414 (17)	0.0558 (6)	
H2	0.8364	0.1990	−0.2022	0.067*	
C3	0.6754 (2)	0.3140 (3)	−0.23634 (18)	0.0665 (7)	
H3	0.6790	0.3436	−0.3058	0.080*	
C4	0.5764 (2)	0.3566 (3)	−0.1955 (2)	0.0692 (7)	
H4	0.5130	0.4163	−0.2374	0.083*	
C5	0.5694 (2)	0.3123 (3)	−0.09323 (19)	0.0651 (7)	
H5	0.5013	0.3405	−0.0665	0.078*	
C6	0.66432 (19)	0.2253 (3)	−0.03029 (17)	0.0529 (6)	
C7	0.86788 (18)	0.0949 (2)	−0.00285 (16)	0.0475 (5)	
H7	0.8684	0.0807	0.0693	0.057*	
C8	0.5779 (2)	0.2404 (4)	0.1251 (2)	0.0907 (9)	
H8A	0.4976	0.1985	0.0925	0.109*	
H8B	0.5762	0.3596	0.1194	0.109*	
C9	0.6111 (2)	0.1903 (3)	0.2398 (2)	0.0626 (7)	
C10	0.7017 (2)	0.2695 (3)	0.3102 (3)	0.0799 (8)	
H10	0.7445	0.3551	0.2866	0.096*	
C11	0.7299 (3)	0.2228 (5)	0.4164 (3)	0.0935 (9)	
H11	0.7907	0.2782	0.4643	0.112*	
C12	0.6691 (4)	0.0959 (5)	0.4514 (2)	0.0952 (10)	
H12	0.6888	0.0640	0.5228	0.114*	
C13	0.5805 (3)	0.0169 (4)	0.3821 (3)	0.0938 (9)	
H13	0.5391	−0.0699	0.4059	0.113*	
C14	0.5506 (2)	0.0633 (3)	0.2766 (2)	0.0742 (8)	
H14	0.4888	0.0081	0.2297	0.089*	
N1	0.95554 (14)	0.0385 (2)	−0.04001 (12)	0.0503 (5)	
O1	0.66609 (13)	0.17497 (19)	0.07219 (12)	0.0690 (5)	

### Atomic displacement parameters (Å^2^)

**Table:**

	*U*^11^	*U*^22^	*U*^33^	*U*^12^	*U*^13^	*U*^23^
C1	0.0451 (12)	0.0464 (13)	0.0445 (12)	0.0027 (10)	0.0074 (10)	0.0012 (10)
C2	0.0595 (14)	0.0573 (14)	0.0508 (14)	0.0035 (12)	0.0126 (12)	0.0019 (11)
C3	0.0826 (18)	0.0636 (16)	0.0512 (14)	0.0121 (14)	0.0092 (14)	0.0112 (12)
C4	0.0698 (17)	0.0688 (17)	0.0615 (16)	0.0169 (13)	−0.0027 (14)	0.0088 (13)
C5	0.0565 (15)	0.0718 (17)	0.0654 (16)	0.0181 (13)	0.0095 (13)	0.0077 (13)
C6	0.0550 (14)	0.0542 (14)	0.0496 (13)	0.0073 (11)	0.0113 (11)	0.0055 (11)
C7	0.0486 (12)	0.0534 (13)	0.0411 (12)	0.0016 (10)	0.0104 (10)	−0.0024 (10)
C8	0.0831 (18)	0.124 (2)	0.0752 (19)	0.0530 (17)	0.0391 (16)	0.0188 (17)
C9	0.0586 (15)	0.0706 (17)	0.0656 (17)	0.0217 (13)	0.0291 (13)	0.0060 (14)
C10	0.0663 (18)	0.080 (2)	0.100 (2)	0.0011 (15)	0.0325 (17)	0.0067 (17)
C11	0.0693 (19)	0.113 (3)	0.092 (2)	0.0163 (18)	0.0034 (18)	−0.022 (2)
C12	0.115 (3)	0.107 (3)	0.069 (2)	0.046 (2)	0.030 (2)	0.0131 (19)
C13	0.123 (3)	0.076 (2)	0.096 (2)	0.0074 (19)	0.053 (2)	0.0160 (18)
C14	0.0758 (18)	0.0684 (18)	0.083 (2)	0.0021 (14)	0.0279 (16)	−0.0083 (15)
N1	0.0446 (10)	0.0636 (12)	0.0415 (10)	0.0078 (9)	0.0067 (8)	−0.0011 (8)
O1	0.0666 (10)	0.0865 (12)	0.0603 (10)	0.0333 (9)	0.0278 (8)	0.0168 (9)

### Geometric parameters (Å, °)

**Table:**

C1—C2	1.388 (3)	C8—C9	1.492 (3)
C1—C6	1.398 (3)	C8—H8A	0.9700
C1—C7	1.454 (3)	C8—H8B	0.9700
C2—C3	1.370 (3)	C9—C10	1.368 (3)
C2—H2	0.9300	C9—C14	1.372 (3)
C3—C4	1.370 (3)	C10—C11	1.382 (4)
C3—H3	0.9300	C10—H10	0.9300
C4—C5	1.376 (3)	C11—C12	1.362 (4)
C4—H4	0.9300	C11—H11	0.9300
C5—C6	1.384 (3)	C12—C13	1.346 (4)
C5—H5	0.9300	C12—H12	0.9300
C6—O1	1.370 (2)	C13—C14	1.373 (4)
C7—N1	1.266 (2)	C13—H13	0.9300
C7—H7	0.9300	C14—H14	0.9300
C8—O1	1.417 (2)	N1—N1^i^	1.414 (3)
			
C2—C1—C6	117.99 (19)	O1—C8—H8B	110.0
C2—C1—C7	121.61 (19)	C9—C8—H8B	110.0
C6—C1—C7	120.36 (18)	H8A—C8—H8B	108.4
C3—C2—C1	121.6 (2)	C10—C9—C14	118.7 (2)
C3—C2—H2	119.2	C10—C9—C8	121.0 (3)
C1—C2—H2	119.2	C14—C9—C8	120.3 (3)
C4—C3—C2	119.5 (2)	C9—C10—C11	120.1 (3)
C4—C3—H3	120.2	C9—C10—H10	119.9
C2—C3—H3	120.2	C11—C10—H10	119.9
C3—C4—C5	120.8 (2)	C12—C11—C10	120.3 (3)
C3—C4—H4	119.6	C12—C11—H11	119.8
C5—C4—H4	119.6	C10—C11—H11	119.8
C4—C5—C6	119.7 (2)	C13—C12—C11	119.7 (3)
C4—C5—H5	120.2	C13—C12—H12	120.1
C6—C5—H5	120.2	C11—C12—H12	120.1
O1—C6—C5	124.2 (2)	C12—C13—C14	120.6 (3)
O1—C6—C1	115.37 (18)	C12—C13—H13	119.7
C5—C6—C1	120.4 (2)	C14—C13—H13	119.7
N1—C7—C1	121.61 (18)	C9—C14—C13	120.6 (3)
N1—C7—H7	119.2	C9—C14—H14	119.7
C1—C7—H7	119.2	C13—C14—H14	119.7
O1—C8—C9	108.35 (19)	C7—N1—N1^i^	111.88 (19)
O1—C8—H8A	110.0	C6—O1—C8	118.49 (17)
C9—C8—H8A	110.0		
			
C6—C1—C2—C3	0.2 (3)	O1—C8—C9—C14	−102.1 (3)
C7—C1—C2—C3	−177.7 (2)	C14—C9—C10—C11	−0.8 (4)
C1—C2—C3—C4	0.1 (3)	C8—C9—C10—C11	179.1 (2)
C2—C3—C4—C5	−0.6 (4)	C9—C10—C11—C12	1.1 (4)
C3—C4—C5—C6	0.8 (4)	C10—C11—C12—C13	−0.6 (4)
C4—C5—C6—O1	−179.8 (2)	C11—C12—C13—C14	−0.1 (4)
C4—C5—C6—C1	−0.5 (3)	C10—C9—C14—C13	0.1 (3)
C2—C1—C6—O1	179.35 (18)	C8—C9—C14—C13	−179.8 (2)
C7—C1—C6—O1	−2.8 (3)	C12—C13—C14—C9	0.4 (4)
C2—C1—C6—C5	0.0 (3)	C1—C7—N1—N1^i^	179.20 (19)
C7—C1—C6—C5	177.9 (2)	C5—C6—O1—C8	−12.4 (3)
C2—C1—C7—N1	−9.7 (3)	C1—C6—O1—C8	168.4 (2)
C6—C1—C7—N1	172.5 (2)	C9—C8—O1—C6	−170.5 (2)
O1—C8—C9—C10	78.1 (3)		

Symmetry codes: (i) −*x*+2, −*y*, −*z*.
